# Vulnerabilities Prompting Use of Technology and Screen by Mothers of Autistic Children in India: Lived Experiences and Comparison to Scientific Literature

**DOI:** 10.1007/s11013-022-09796-z

**Published:** 2022-08-20

**Authors:** Seema Girija Lal, Elena Syurina, Laura Pilz González, Esmée L. S. Bally, Vandana Gopikumar, J. G. F. Bunders-Aelen

**Affiliations:** 1 Faculty of Science, Athena Institute, Vrije Universiteit Amsterdam, Amsterdam, The Netherlands; 2 Together We Can, Kochi, Kerala India; 3 The Banyan Academy of Leadership in Mental Health, Chennai, India; 4 Charité –Universitätsmedizin Berlin, Corporate Member of Freie Universität Berlin and Humboldt-Universität Zu Berlin, Institute of Health and Nursing Science, Augustenburger Platz 1, 13353 Berlin, Germany

**Keywords:** Mother, Vulnerability, Autism, Influence, Technology, Screen, Time

## Abstract

Technology and screen media has its place in every home, yet the influences of the same are less known. This research aims to explore the vulnerabilities that prompt the mothers to use screen media for their children, prior to a diagnosis of autism for their child. It also aims to explore literature the influence of screen media on speech and language development in children. This study combined semi-structured interviews with 16 mothers of autistic children in Southern India and a scoping literature review that resulted in 24 articles. The literature refers to a positive influence when co-viewing with the child, and it predominantly highlights improvements in speech and not in language. The interviews revealed that screens were used as a means of support, a language and learning development tool, or as a calming technique. Thus, the study shows that the mothers resorted to screen use for their children more out of helplessness, and not as an informed choice. Mothers of autistic children clearly express their vulnerabilities and indicate feelings of being lost without advice, with regard to use of screen-time. This suggests a need for more research into how they can be supported.

## Introduction

Autism spectrum disorder (ASD) is one of the fastest-growing childhood neurodevelopmental disorders across the world, with an exponential spurt in growth during the past 20 years (Maenner, Shaw, and Baio 2020). Despite the lack of evidence available on the prevalence of ASD in the Indian context, this condition is estimated to affect over two million people in the country. Nevertheless, this number has been shown to be most likely an underrepresentation of the true prevalence of ASD (Raina et al. 2017; Rudra et al. 2017).

ASD mainly affects areas regarding social interaction, verbal and nonverbal communication and the development of social and language milestones. (APA 2013; Baishya et al. 2018). Such effects are often not detected until at least a year after birth. And so they are not called out as matters of concern at the time of birth by healthcare professionals or by significant members in one’s family. (Ozonoff et al. 2018). Differences in social, emotional and language development in the child are noticed only when they are significantly deviant or delayed from available childhood developmental checklists (Koh, et.al 2016). But this usually does not happen before the child reaches one year of age (Flensborg-Madsen, Grønkjær, and Mortensen 2019).

It has been long known that the journey of a mother of a child who has not yet been diagnosed with ASD is one of the most challenging ones. The process of getting an assessment or diagnosis is in itself a prolonged, trying and disheartening process. And this process is only the beginning of the exploration into the uncharted facets of life ahead (Mulligan et al. 2012). Soon after a diagnosis, mothers of autistic children go through an intensely emotional phase that is full of unanswered questions such as *“why me?”* or *“what did I do to cause this?”*, and prompt them to understand more about the diagnosis and the causes of ASD. A recent qualitative study of lived experiences of mothers of autistic children shows the significance of understanding the vulnerabilities experienced by the mothers due to their emotional, social and family burden. (Papadopoulos, (2021). Another study shows how mothers, who have recognised early signs, experience delay in getting a diagnosis and initiating intervention (Shattnawi et al. 2021).

Despite the relatively low number of studies on the family context of autistic children in India, there is substantial evidence stating that having an autistic child can lead to significant physical, psychological, social and environmental impairments in the functioning of the family (Das et al. 2017). Such effects are even more prominent among mothers as they are usually the primary caregivers in Indian households, which in turn highlights their need for social and emotional support (Singh, Ghosh, and Nandi 2017).

The use of screen and technology as a potential support medium in general, and specifically in the early years of a child’s life, is one of the most debated areas in clinical practice within the Indian context (Kardaras 2016; Salame et al. 2020).

Some studies associate intensive early screen exposure to negative effects on a child’s attention span, language development, emotional regulation and socialisation (Sadeghi et al. 2019). There have also been studies in the field of ASD showing improvement in symptoms after the parents stop screen exposure for a few months. And it has often been recommended to restrict screen-time in families of autistic children (Harlé 2019; Krupa et al. 2019). Depending on the type of content consumed, the use of screen media as a learning tool has been shown to have an effect on the child (Greenhow and Askari 2017). This conflict in opinions can be very confusing for mothers of autistic children and thus further affect their vulnerabilities and uncertainties. The issue of giving support to mothers with autistic children is a very complex one, because of the context in which these mothers and children live.

Culture has a serious impact on the support given, and barriers experienced, as well as on the solutions chosen to overcome these vulnerabilities and uncertainties. This study focuses on the experiences of the mothers within the Indian context, which is characterised particularly by experiences of sociocultural dynamics regarding patriarchal family structures, stigma and legislation. In the Indian context of strong familial ties, the extended family can provide much-needed social, cultural and financial support, as well as contribute to marginalization through expressions of pervasive stigma related to mental health. This stigma is also largely influenced by the lack of clear estimates of the prevalence of autism in India and the resulting gap in knowledge, awareness and appropriate care (Mahomed et al. 2019; Jagan and Sathiyaseelan, 2016).

To provide mothers with the support they need, it is important to review the source of their vulnerabilities, and their lived experiences. We aim to gain a better understanding of their struggles by looking at their experiences and opinions regarding screen use before and after their child's ASD diagnosis, and by illustrating perceptions about the content viewed, and the reasons why they used screen media. We also expect to provide some clarity to health professionals on how to counsel parents, specifically mothers, regarding screen-time use in their homes.

## Method

This research is a sub-study of a broader qualitative research project on factors contributing to the empowerment of mothers with autistic children in India. During this project, the focus was on the collection and analysis of the lived experiences of mothers, from the birth of their child to the present day. This particular study zooms in on only one aspect of this journey: screen use. During the interview data collection process, it was noted that there is likely to be confusion and insecurity among mothers about the use of technology. This prompted the need to investigate the link between the Evidence-Based Medicine (EBM) knowledge, the transfer of this knowledge to the mothers, and the real-life factors that influence the use of technology. The resulting study consisted of two sequential research branches: (i) the interviews with mothers, whose children were diagnosed with ASD and (ii) an additional review of current scientific literature.

### Phase 1: Qualitative Interviews with Mothers

The qualitative data collection was conducted in two steps. First, initial interviews were conducted to understand what empowers the mothers, and overcome their vulnerabilities. As previously mentioned, one of the main themes that emerged in this pilot study was the use of technology. Consequently, in the 15 semi-structured interviews that followed, the mothers were specifically asked about their perceptions on the use of technology during the phase from childbirth to getting their child’s ASD diagnosis. The total sample size of the actual interviews was 16 mothers of which one was from the initial interview set (the exploratory phase). This interview was added from the exploratory phase because this mother had voiced her views on screen and technology use strongly, but was unable to participate in the semi-structured interview. All other mothers who voiced the same concern in the initial set of interviews were included for the in-depth study as well.

### Demographic Details

Prior to collecting data, ethical approval was received from The BALM, Chennai (The Banyan Academy of Leadership in Mental Health), which has an independent ethical committee. During the study, explicit informed consent was obtained from each participant, in either a written or oral form, before any form of data collection. The participants were informed about the aim and procedures of the research, its implications, their rights as participants, and the fact that participation was entirely voluntary and could be stopped at any point in time.

#### Data Collection

A poster was made inviting participation in the research and was circulated mainly via social media. The only criterion was that the participant be a mother of a child with autism in India. From there on, a snowballing technique for the further participant recruitment was used. The participants were given a choice on how they wanted the interview to take place: face to face, over the telephone, or over WhatsApp voice records. All 16 participants chose telephone interviews.

The interviews were semi-structured lasting 45 min to an hour, using open-ended questions with a focus on the participants' lived experiences and perceptions. The following questions were included:

- Did you use any screen media or technology for your child before getting the diagnosis?
- What kind of screen media was used and why?
- Did you find screen media helpful?
- Did you find any influence of screen media in your child’s development?
- What made you use the screen media?

The telephonic conversations were recorded using a call recorder application and were later transcribed verbatim. The completed transcripts were checked against the recordings for accuracy with additional focus on changes in voice and tones.

#### Data Analysis

All the interviews were professionally transcribed and checked for accuracy by the first author. The thematic analysis roughly followed the six-phase framework of Braun and Clarke (2006) to identify key patterns in the data. The process began with a familiarization of the data through reading and re-reading the transcripts (predominantly undertaken by the first author and supported by the second and third authors). This was followed by the generation of initial codes relevant to the core area of the study, namely, the mother’s perceptions on the influences of screen media on their child, prior to the child's ASD diagnosis. Subsequently, three broad codes were arrived at by the first three authors. These were the mothers’ perceptions of positive, negative and neutral influences of screens on their children. The next step in the analysis, undertaken by the first and third authors, was to find the relevant themes and sub-themes within these codes. These themes were clustered by the first three authors and categorized as follows: types of screens used, reasons of screen use, influence of screens on language and learning development of the child, type of content viewed and perceived consequences of screen use. These themes were then checked by a peer researcher by linking them to the initial codes. Discrepancies were discussed and a consensus arrived at. The key theme that emerged was a perceived positive influence on language and learning, on which the second and fourth authors conducted an extensive literature review. All authors were involved in an inductive process to arrive at key themes as they did not rely on an existing framework to interpret the data. This gave scope for new knowledge to be created within less researched fields of study and in this case the vulnerabilities of mothers prompting them to use screens for young children and the uncertainties surrounding its impact in both scientific literature and practice. The first author is a psychologist and founder of an advocacy group networking with mothers of autistic children, the second author is a neuro-psychologist, the third and fourth authors are research assistants and the fifth and sixth authors are mental health scholars. The peer researcher is a post doc student studying the concept of vulnerability.

### Phase 2: Literature Review

Since many mothers mentioned that they were confused about the use of technology when parenting, a systematic search of scientific literature was conducted to explore the positive and negative effects of screen media on children's language development.

The literature search was conducted in November 2018 using the following three main databases: PubMed, EMBASE and PsycINFO. The search syntax included following terms: "screen media'', "information technology", "mobile device”, ''video” as well as synonyms for these terms in combination with "language development", "speech development", "communication" and related terms. These search combinations were then connected to the study population by including keywords such as "young children'', "infants" or "pre-schoolers". Different combinations including search terms, MeSH terms and phrases of these three "categories" were made.

### Study Selection

**Fig. 1:**
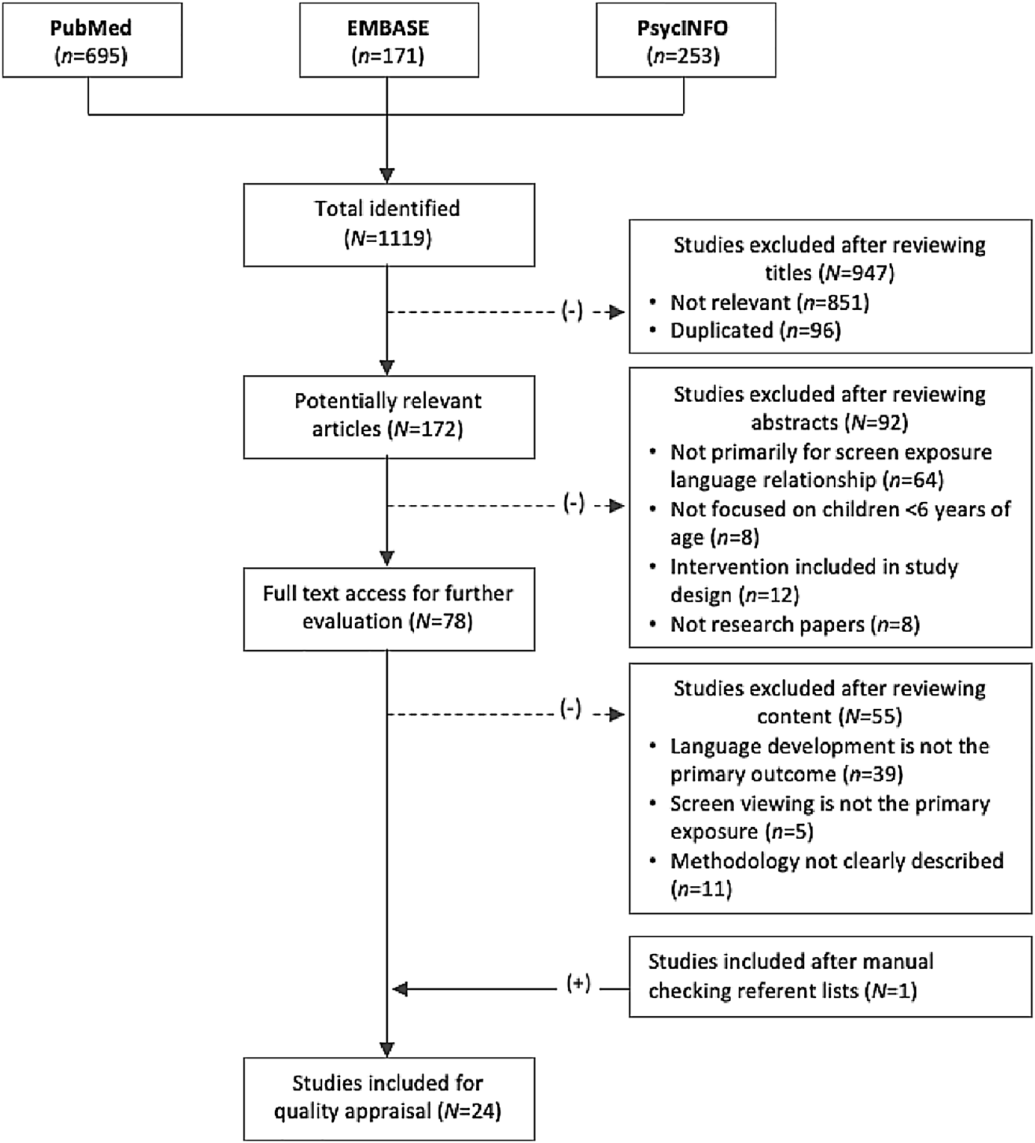
Selection process of included studies

Identified studies were assessed based on the inclusion and exclusion criteria as shown in (Table 1). The database search resulted in 1119 publications. After the removal of duplicates, the titles and abstracts were screened. The full text of 78 articles was obtained for further review. This screening resulted in 23 articles meeting the inclusion criteria. In addition, one article was obtained through reference list searches of articles specifically focussing on the relationship between exposure to screen media and symptoms of ASD. The full selection process can be seen in (Fig. 1).

**Table 1:** Inclusion and exclusion criteria in the appraisal phase

Inclusion		
Category	Criteria	Rationale for inclusion
Language	English	Mastered by author and common language among scientific publications
Type of publication	Peer-reviewed scientific article	Most reliable form of research dissemination (Pöschl 2012). Authors are obliged to meet the high standards set by peers in their discipline
Time period	January 2008–November 2018	Literature on technology use by young children emerged increasingly in recent years
Characteristics article	Full text available	Full text is needed to assess an article on quality, including design, study population, methods, etc

Exclusion		
Category	Criteria	Rationale for exclusion
Language	Article not provided in English	–
Type of publication	Grey literature or research pending for publication	–
Time period	Articles published before 2008	–
Characteristics article	Article not available after request	–
Content article	- Article has an intervention design - Subjects (study population) older than 6 years of age - Article focussed on cognitive and social development	Articles with an intervention design alter the association between screen media and language development in children instead of presenting the current effects of screen media on children and are therefore excluded

### Data Extraction and Analysis

An extraction table was used to assist the analysis of selected articles (Table 2). The extraction table contained several categories that helped with seeing similarities and conflicts between publications, and also to have an overview of all articles included. Subsequently, studies were grouped by the associations found (positive, negative, no evidence) and the age of the target study population (younger than 3 years or 3 years and older), in order to identify patterns across the paediatric age spectrum. The newly formed categories provided information on the kind of association or effect found, and the age of the sample included. Due to their scarcity, articles including research on ASD in relation to screen media exposure and language development were analysed separately.

**Table 2:** Categories and units in the extraction table

General	Publication title
	First author
	Year of publication
	Country of study
Research aim and focus	Aim
	Type of screen media exposure
	Target study population
Sample	Sample size
	% of sample female
	Mean age in months
Methodology	Study design
	Study methods
	Predictor variable Confounding factors
	Outcome variable
Findings	Association found Main conclusions
	Negative association (yes/no)
	Positive association (yes/no)

To ensure the integration of different findings across multiple articles, a thematic content analysis was conducted with the support of a qualitative research program (MAXQDA). First, a deductive coding method allowed us to see how the effect was established in one of the three categories: positive effect, negative effect or no effect. The type of screen media exposure was also coded. Second, an inductive approach was used to explore relevant concepts that triggered the effect and so needed to be analysed in a broader perspective.

## Results

### Qualitative Interview Findings

This section describes the essence, themes and sub-themes that emerged from the analysis of the experiences and perceptions of mothers of autistic children, about the influence of technology and various types of screen on their children, before they got the diagnosis of ASD. The general demographics of the mothers who participated can be seen in Table 3.

**Table 3:** Demographic details about participating mothers

Total number of respondents	16 (All mothers had one child with autism)
Educational qualification of the mother	Graduates—n = 9 Post -graduates—n = 7
Gender of autistic child	Boys—n = 14 Girls—n = 2
Year of birth of autistic child	< 2000—n = 2 2000–2010—n = 13 2012—n = 1
Ordinal position of sutistic child	First born—n = 9 Second born—n = 7
Age of diagnosis of ASD	Before 3 years—n = 11 After 3 years below 5—n = 5
Type of family	Nuclear—n = 15 Joint—n = 1
Place of residence	Kerala—n = 12 Karnataka—n = 2 Tamil Nadu—n = 1 Maharashtra—n = 1

### Type of Screens and Content Used

During the interviews, several types of screen devices and their use were mentioned. Among the most common ones were television and mobile phones (smartphones). Regarding the type of content, they mostly mentioned television advertisements, music videos, educational DVDs and cartoons.

### Intention Behind Providing the Screen

From the interviews, it became evident that there are several ways in which a child becomes exposed to the influence of screen devices. The child could have passive exposure to screens, when they are exposed without the parent intending to provide the child with a screen. Or the child could have active exposure to the screens, where the parent intentionally provides a screen to keep them engaged. Thus, the intent behind the child getting exposed to screens and their level of active engagement varies.

Only three mothers out of sixteen stated that the child was unintentionally exposed to screen mediums; it was just a coincidence that the television was on when the child was in the room. The mothers mentioned that the child initially started watching television because the parents were doing so. In such cases, they did not recognize screen use as something out of routine as the screens were part of their daily lives.

"They (referring to her children) used to watch the ‘Thomas the Train’ cartoon together. He (referring to autistic child) would come in front and watch it with great interest, laughing and playing…When he (referring to the husband) would come back home, he would watch TV instead of being with the children, so ____- (mentions the name of the child) would also do the same."

On the contrary, other mothers mentioned that they purposely provided their child with screens: to either distract them, or to help them learn. Here, it is noted that the use of screen devices as a distraction tool was an intentional decision of the mothers. While some mothers indeed relied on screens simply to distract and entertain their child, others took a more engaging and interactive approach, by sitting with the child as they used the screen.

"I used to sit with him (referring to the child) and I would literally be explaining what is happening, to make it make more interactive. But if you ask me, yes! He had screen time, and he had a lot of screen time, but he preferred it and he was attracted to it"

### Reasons for Screen Use

In general, the main reasons for the use of screen devices were to calm the child, to use it as a tool for distraction, or as a learning tool for the child. These reasons are often co-existent in the daily routines of the family environment.

Eleven mothers out of the sixteen mentioned that they used screens due to lack of time and energy, and the support of others in their daily tasks. Even if the mothers themselves believed that screen exposure was not positive for the child in the long run, they found themselves with no other tools in certain situations. For instance, when they still needed to finish work they often felt forced to allow their child screen-time.

"Most of the mothers satisfy the child's primary needs and then have to get back to their own household work or professional work. (…) I also did it (use screens), basically to finish off my work, especially when there is no support."

Additionally, the use of screen media helped mothers handle challenging situations that are often linked to social engagements. In such cases, the use of screen devices happened when alternative measures were either more complicated or seemed ineffective. The mothers resorted to giving their child a screen, for example, their smartphone, to either calm them down or to distract them.

Nine mothers mentioned that they felt the use of screen exposure was an optimal way to engage their child to learn, as well as to stimulate their interest in music. Here, two different modes of using technology as a learning tool could be identified. The first one was as an actual resource for learning, and the second one as a positive reinforcement.

"He (referring to the child) would not listen to what we say but he would listen to the TV advertisements. (…) He even learnt to clap hands by watching TV. Then, he started to imitate more things when we used the gadget as a reward. We will ask him to imitate something and if he did it, we would give (him) the gadget."

#### Screen Use as a Learning Resource

As mentioned above, there was a common belief that the use of technology resulted in the positive reinforcement of learning. While several areas of learning were addressed with the use of screens as a tool, the most common one stated was the language development of the child. Fourteen mothers mentioned that they used either special video materials, such as educational DVDs, or even common commercials in the attempt to expand the vocabulary of the child as well as to increase and improve their speech production.

"I used TV as a learning resource. (…) The kid is not interested in real life, why can't we use his interest in TV into making him learn?"

One of the mothers mentioned that she used the television as a resource to teach her child to perform daily activities such as brushing their teeth or washing their hair. She noted that she had difficulties in explaining abstract skills to her child, and managed to do so by using commercials in which people were performing said actions. Similar to this case, the use of screen devices for educational purposes helped the child learn through a process of imitation. These imitations went so far that in one case, a mother mentioned that her child was able to perfectly imitate the dialect and speech used in television. This ability declined and even disappeared after television viewing was excluded from the child's life.

### Increase in the Use of Screens and Perceived Consequences

Seven mothers mentioned that they did not perceive screen use as a negative exposure, as it was part of their daily routine in the household. The exposure was not just for the child alone, but also for the rest of the family. While it was shown that some children are more resilient to possible effects of screen use, others were more prone to them. Among the most commonly perceived adverse effects of the use of screen was the increasing dependency of the child to the screen. In six cases, this was even described by the mothers as a form of addiction.

"He wanted only that (gadgets) and started crying non-stop. When he didn't stop for half an hour, I was afraid and phoned my husband and said: I don't know why this child is crying for so long! That day, he cried for about one and half hours! It was on that day that we first noticed it (his addiction). He was really stubborn, and he only wanted that…. He wanted more gadgets."

At first, the mothers did not think that the exposure to the television was worrisome. However, this became evident to them later, when they saw the change in the child’s behaviour when the access to television was stopped abruptly. Two mothers did not realize their child's strong need to have screens as a support, until they noticed unexpected and negative behaviour in the child, when screen-time and technology use was reduced. The mothers described such behaviour as having difficulty in getting the child to calm down or focus without the use of screens, and the child crying for very long periods of time as well as throwing tantrums when the screen was turned off. Such new or unusual behaviour resulted in three mothers seeking professional help.

"When she (referring to the child) had the chickenpox she was full time in front of the TV as she could not do anything else. And after she recovered, we noticed a sudden change in her behaviour, she would not stop crying if we turned the TV off. That is when we took her to the doctor for consultation."

### Insecurity and Inner Conflict (Explicit and Implicit)

While all mothers mentioned their child's screen use and access to technology via different types of screens, fourteen of them said they regretted their excessive use, in retrospect. All of them mentioned the importance of being aware and informed of the possible effects of the use of technology and screen-time on children.

"I believe children nowadays are more addicted so mothers must know about the harmful effects (of technology)."

There were two mothers who perceived screen use as entirely positive, and felt unsure about restraining their use. Although there were some differences in opinions in this regard, there was a general insecurity and confusion on whether the influence of screen use was indeed of a positive or negative nature.

"I don't know about that … I don't know if it was good or bad. I can only say it may be possible, because I don't know much, I don't know if it is right or wrong."

However, when reflecting on their experiences, mothers stated that they did not know any other mechanisms to deal with stressful situations then. Twelve mothers mentioned that they were judged by their relatives and they were told that it was because of the use of screens that their child was autistic. Regardless of the perceived external judgement from their social environment, the mothers mentioned that they would not have known how to cope with the lack of support, the pressure on them, and their role as mothers of autistic children.

"Yes, I also thought, when we go somewhere, I need to talk to relatives and enjoy and so engage him in this... When we see the child's behaviour like this (inability to sit and play along with others) in front of others, we feel like we have gone a bit down in their eyes. So, he will not make any other issues. This is wrong, this is not right. That was all wrong. If we had dealt with the child without all these (gadgets and screens), it would have been better."

When reflecting on their experiences, fifteen of them mentioned that they would recommend future mothers to limit the use of screen devices or to find a balance. For instance, all mothers recommended an intermediate approach to the use of screens, where social interactions are also actively pursued, along with the use of technology. They saw this as an intermediate approach, as they could not realistically foresee a reduction of their child's use of, and exposure to technology.

### Literature Review

Out of the 24 studies included, ten reviews mentioned a positive association between exposure to screen media and language development (Ferguson and Donnellan 2014; Kirkorian, Choi, and Pempek 2016; Lee, Spence, and Carson 2017; Lytle, Garcia-Sierra, and Kuhl 2018; Myers et al. 2017; Roseberry et al. 2009, Roseberry, Hirsh-Pasek, and Golinkoff 2014; Scofield and Williams 2009; Strouse et al. 2018; O’Doherty et al. 2011). These studies highlighted the importance of social interaction in the use of screen devices. Co-viewing with a parent and even video chats were highlighted as viable options in which the exposure to screen goes hand in hand with social interaction. However, none of the studies presented an influence of screen use on receptive language, but only on expressive language, which is the production of the sounds or repeating without understanding the meaning (Ferguson and Donnellan 2014).

The other ten studies showed a negative influence and mentioned the risk of a language delay in proportion with more hours of daily screen-time (Byeon and Hong 2015; Chonchaiya and Pruksananonda 2008; Christakis et al. 2009; Collet et al. 2019; Duch et al. 2013; Hermawati, et al,. 2018; Lin et al. 2015; Okuma and Tanimura 2009; Tomopoulos et al. 2010; Zimmerman et al. 2009). These studies specifically indicated that higher use of screen devices reinforces shorter attention spans and hyperactivity. It was also mentioned that screens inhibit conversational skills.

The remaining four studies did not mention any influence or effect of screen media on the language development of children (DeLoache et al. 2010; Robb, Richert, and Wartella 2009; Taylor, Monaghan, and Westermann 2018; Richert et al. 2010). Instead, one of the studies highlighted that only a few of the words featured in the screen device were indeed learned, even with a substantial amount of exposure (DeLoache et al. 2010; Robb, Richert, and Wartella 2009). Along with this, one of the studies even concluded that screen media has no real impact on the language and vocabulary size of children (Taylor, Monaghan, and Westermann 2018,2018). These results are somewhat counterintuitive in relation to the other studies found.

## Discussion

In this paper, the authors explore the various challenges that mothers of autistic children underwent during the period between childbirth and the diagnosis of ASD of their child. While looking at each mothers' experiential journey, the influence of screen media stood out. The multiple reasons why mothers tend to use screen media with their children and their experiences with it are presented in this study.

In line with additional literature on parenting autistic children, a blend of traditional, modern and creative resources, such as the use of technology, to manage and cope with aspects of their child’s disability, is highlighted (John and Roblyer 2017). This paper shows that one of the biggest reasons why mothers tend to use screen media is the pervasive sense of helplessness all through their journey from childbirth to the diagnosis of ASD of their child. The lack of awareness about how screen media influences a child's development and the scarcity of "better" alternatives stand out as crucial points in the increased use of screen media. This lack of awareness is compounded by the vulnerability that women face, especially when being mothers of autistic children. These two aspects prevent the maternal agency from taking action for their child's health.

The additional literature review that was conducted allowed us to compare the results from the interviews with the broader body of evidence on this topic. Overall, the literature review did not show any conclusive evidence of either positive or negative effects of technology or screen media on children. Positive influences concerning language development were mostly about the enrichment of the child's vocabulary. However, the use of screen media was shown to not have any impact on the social communication skills among children, which is a primary deficit in ASD (Ferguson and Donnellan 2014; Zimmerman et al. 2009). Additionally, the conduction of the literature review highlighted that the limited evidence available predominantly focuses on Western countries. This gap in knowledge was even more profound on the experiences of ASD and screen use.

The aforementioned divergence in opinions and evidence on the effects of technology or social media on autistic children was also prevalent in the interviews. While parent’s help-seeking behaviour and coping mechanisms are strongly influenced by their beliefs about the disorder and their expectations about their child’s future, often, in retrospect, they realize the futility of their efforts too late, as they see no real results from them (Juneja and Sairam 2018). This was also seen when mothers spoke about positive influences during their lived experience. However, in retrospect they spoke about screen-time negatively. In line with the latter aspect, they advised future mothers to keep their children away from screens and technology. In a broader sense, it meant that during their lived experiences, a lot of mothers did not see alternatives, but in hindsight, they regretted their decisions. Even though they do not see it as a cause of ASD, they did feel there was an influence.

Mothers linked the use of screen media to not only individual experiences, but also to broader family dynamics. They, therefore, mentioned relying on the use of screen media to get daily routines with their child going and to be able to perform specific actions such as sitting their child down, feeding them, or socially interacting with them, and as a teaching or learning tool. In line with this, Desai et al. (2012) highlight parents' and especially mothers' fundamental concerns when taking care of a child with ASD. Such concerns relate to learning to meet new and unfamiliar challenges, caring for their child's basic needs, as well as finding ways to constructively engage with their children. (Desai et al. 2012).

The interviewed mothers also mentioned that the lack of support they experienced in taking care of their child and their household was a reason for the increased use of technology devices to cope with their child. And for most of them, their professional work added to their responsibilities. While they highlighted multiple times that they were unsure about the possible effects of screen media on their child, they still relied on this option out of a lack of better means to cope with their child. Six of them even blamed themselves or their spouses while reflecting on their journey. Literature dealing with the vulnerability of parents of autistic children highlights the feelings of helplessness and uncertainty of parents when taking care of their child with ASD (Depape and Lindsay 2015). Indeed, becoming a parent calls for a change in roles that can be identified as a crisis. However, the birth and diagnosis of an autistic child adds a situational crisis that goes beyond the generic parental roles. Especially for mothers, this change in role is even more drastic and traumatic, as the images and expectations of motherhood constructed by society are based on "healthy" children (Vidyasagar and Koshy 2010). In line with this, some mothers mentioned how they did not want to be judged as a "bad mother" for not being able to manage their own child, and hence resorted to screen media as a coping mechanism during social interactions or gatherings.

The majority of the interviewed mothers laid stress on informing new mothers about the importance of knowing the possible effects that screen media has on their child. Similarly to the results of the literature review, mothers who mentioned positive influence were all talking about co-viewing or using it with ample social interaction as well. Additionally, eleven of them reached for the use of technology as an almost desperate attempt to find a solution as the child was not responding to conventional methods of teaching and learning. This clearly shows a lack of proper guidance on how best to work with the child and not knowing when to seek professional help. Nevertheless, proper structures or information sources that can support families and can be translated to the Indian context are missing (Juneja and Sairam 2018; Desai et al. 2012; Divan et al. 2012). Parenting and motherhood sharply differ depending on their societal and environmental context. For instance, Indian parenting style is starkly different from that of the Western hemisphere (John and Roblyer 2017). When reviewing and utilizing the findings of this study, it is important to bear in mind the sociocultural context in which it was conducted. Among others things, the Indian context is characterised by the lack of support for parents, and especially mothers as the primary caregivers of autistic children. This lack of support prevents mothers from fulfilling their maternal role in a supportive and informed way. This lone caregiving by the mother occurs in the context of shared patriarchal parenting that is typical for the Indian family dynamics and context (John and Roblyer 2017). As shown through the experiences of mothers, they end up taking their own measures that they think are best for their child in the given situational context.

Within this study, we have reviewed maternal experiences and opinions through a retrospective lens. Fifteen of the mothers did say that their opinion and vision of the use of screens did change over time. It would be interesting to see if there are significant differences in the attitudes among the mothers of children of different age categories, i.e. do mothers of younger children feel more or less relaxed about the use of screens. Due to the relatively limited sample size, we were unable to perform such analysis within the present work. All of the above is indicative that we need more research on the effects of screens on young children. We also need more research into the sources of vulnerabilities of mothers of autistic children, and on how they can be addressed. The participating mothers of this study did not have enough knowledge about the influence of screen media, which was reflected in both their perceptions and practices. Their, sometimes desperate, turn to social media in order to cope with the lack of support—in raising their children, maintaining a home and being professionals—highlights the urgent need for support not only within the family, but also within the health structure.

This is especially true during the lockdown measures related to the COVID-19 pandemic and the move to online education and work. Parents were encouraged to set up supervised peer video chats, and even some preschools moved online (Mental Health and Coping during COVID-19 | CDC, n.d.). Indeed, it has since been demonstrated that technology can help children learn and comprehend stories even at preschool age (Gaudreau et al. 2020). Furthermore, Cristia et al. (2017) notes how the use of technology, rather than being an influence in and of itself, is an integrated system of influence involving parents, content developers, educators, community culture, incentive structures and events. This coupling of research and recent events indicates a need for a paradigm shift in how researchers approach the question of technology use by children with autism. There needs to be a shift away from a linear positive/negative outcome, and towards a dynamic understanding of how to achieve positive effects. The question becomes less about whether we should use technology and more about how we can go about using it safely and productively. Future research should therefore address the issue of empowering parents, whatever their background, to use technology in an informed manner. We need policies and guidelines for professionals to focus more on empowering mothers and thereby their families and children.

## Conclusion

The ultimate aim of the study was to gain a better understanding of the vulnerabilities of the mothers through their lived experiences with the use of screen and technology before their child was diagnosed with ASD. It was clear from the interviews that the mothers did not have a lot of information or support when caring for their child. This resulted in stressful and uncomfortable situations in which the easiest methods, such as the use of technology to distract their children, were taken to protect themselves. Making matters even more confusing for them is the absence of clear scientific guidance on the proper use of technology. This lack of evidence prevents mothers from making informed decisions around the topic. The general advice given by both, the interviewed mothers and the available literature on the topic, has been to actively participate and co-watch when using screen media with a child. However, from the interviews, it was clear that this was done only in a few cases. The reasons for this are (i) the aforementioned lack of knowledge and opportunity for informed decision making, and (ii) the vulnerable position in which the mothers find themselves. Most mothers in retrospect regretted their decision of having used screen-time the way they did, and their advice to future mothers was to use it more wisely. It is therefore highly recommended that there should be more studies specifically to assess the influence of various types of screen exposure in children below 6 years.

With the COVID pandemic and the thrust towards using screens and technology as a means for education, entertainment and engagement, the need for more support for mothers to make informed decisions regarding technology use has become more evident. The conclusion of this study is not regarding technology or no technology, but more about how it is used. What is seen is that technology is only a tool and its efficacy depends on how it is used. What is seen in the literature is that human contact and context are important.
